# Development and Validation of RP-HPLC Method for Simultaneous Estimation of Atorvastatin Calcium and Fenofibrate in Tablet Dosage Forms

**DOI:** 10.4103/0250-474X.41473

**Published:** 2008

**Authors:** N. Jain, R. Raghuwanshi, Deeti Jain

**Affiliations:** School of Pharmaceutical Sciences, Rajiv Gandhi Proudhyogiki Vishwavidhyalaya, Bhopal-462 036, India

**Keywords:** RP-HPLC, simultaneous determination, atorvastatin, calcium, fenofibrate, development and validation

## Abstract

A reverse phase high performance liquid chromatographic method was developed for the simultaneous estimation of atorvastatin calcium and fenofibrate in tablet formulation. The separation was achieved by Luna C18 column and methanol:acetate buffer pH 3.7 (82:18 v/v) as mobile phase, at a flow rate of 1.5 ml/min. Detection was carried out at 248 nm. Retention time of atorvastatin calcium and fenofibrate was found to be 3.02+0.1 and 9.05+0.2 min, respectively. The method has been validated for linearity, accuracy and precision. Linearity for atorvastatin calcium and Fenofibrate were in the range of 1-5 μg/ml and 16-80 μg/ml, respectively. The mean recoveries obtained for Atorvastatin calcium and fenofibrate were 101.76% and 100.06%, respectively. Developed method was found to be accurate, precise, selective and rapid for simultaneous estimation of atorvastatin calcium and fenofibrate in tablets.

Atorvastatin calcium^1^,^2^ (AC) is (β R, δ R)-2-(4-fluorophenyl)-β,δ-dihydroxy-5-(1-methyl ethyl)-3-phenyl-4-((phenyl amino)carbonyl)-1H-pyrrole-1-hepatonoic acid, a HMG CoA reductase inhibitors and fenofibrate 3-9 (FB) is 2-[4-(4-chlorobenzoyl)phenoxy]-2-methyl-propanoic acid, 1-methylethyl ester. It is indicated for the treatment of hypercholesterolemia and mixed dyslipidemia^11^. Tablet formulation containing 10 mg of AC and 200 mg of FB is available (Lorilip Micro Labs. Ltd., Pondicherry, Tornet-TG Lupin LTD, Mumbai). The survey of literature revealed some HPLC methods for determination of AC and FB in tablet separately and a simultaneous method for estimation of AC and aspirin in their combined dosage form by HPLC. A high performance liquid chromatographic method for determination of fenofibrate in tablets is also reported. However, no HPLC method for the simultaneous estimation of atorvastatin calcium and fenofibrate in combined dosage forms has been reported so far^12^. The present work describes the development of simple, precise and accurate isocratic reverse phase HPLC method for simultaneous estimation of AC and FB in tablets.

The drug sample, AC and FB were obtained as gift samples from the Ranbaxy Labs Ltd, Dewas, anhydrous sodium acetate AR and glacial acetic acid AR were purchased from Merck Chemical Division Ltd., Mumbai. Triple distilled water was used for analysis.

A gradient high pressure liquid chromatograph (Shimadzu HPLC class VP series) with two LC-10ATVp pumps, variable wavelength programmable UV/Vis detector SPD-10AVp, SCL-10AVp system controller (Shimadzu) and operating software Shimadzu class VP version 6.12 SPz data station was used. The chromatography column used was a reverse phase luna C_18_ column (250×4.6 mm i.d. particle size 5 μ). A mixture of methanol and acetate buffer pH 3.7 in the ratio of 82:18% v/v was used as mobile phase and was filtered before use through 0.45 μ membrane filter. The flow rate of the mobile phase was maintained at 1.5 ml/min. Detection was carried out at 248 nm at 25°.

Standard stock solution of AC and FB (1000 μg/ml) was prepared in a mixture of methanol and water (80:20 v/v) as diluent separately. The standard solutions were further diluted to contain a mixture of 32 μg/ml of AC and 2 μg/ml of FB. Twenty tablets of Lorilip (Micro Labs. Ltd., Pondicherry) and Tornet -TG (Lupin Ltd., Mumbai) each containing 10 mg of AC and 160 mg of FB were weighed and finely powered separately. Powder equivalent to 10 mg of AC and 160 mg of FB was weighed and transferred to a sintered glass crucible and drug was extracted with three 20 ml quantities of mixture of diluent. The combined extracts were made up to 100 ml and further dilutions were made to get a concentration of 32 μg/ml of AC and 2 μg/ml of FB. The contents were mixed thoroughly and filtered through a 0.45 μ filter. Twenty microlitres of the test and standard solutions were injected separately and chromatogram was recorded.

The present investigation was aimed at developing a simple, precise and accurate HPLC method to estimate AC and FB in tablets using the widely used RP-HPLC C_18_ column (Luna). The mobile phase was optimized with methanol and acetate buffer pH 3.7 in proportions of 82:18 v/v. With the above mobile phase a good resolution between AC and FB was achieved with a reasonably short runtime of 10 min. The criteria employed for assessing the suitability of above said solvent system were cost, time required for analysis, solvent noise, preparatory steps involved and the use of same solvent system for the extraction of drug from the formulation excipient matrix for the estimation of drug content. UV detection was carried out at 248 nm as AC and FB both showed good absorbance at this wavelength.

The retention time of AC and FB was found to be 3.02±0.1 and 9.05±0.2 min, respectively. The capacity factor (k′ ) of AC and FB was found to be 0.67 and 4.07, respectively. The peak shapes of both the drugs were symmetrical and the asymmetry factor was lesser than 2.0. The proposed method was validated as per the standard analytical procedures. Each of the samples was repeated 6 times and the same retention time was observed in all the cases. Precision of proposed HPLC method was found to be 0.0345 (RSD) for AC and 0.0079 for FB that indicates good precision of the samples analyzed. Linearity experiments were performed by giving six replicate for both the components and the response was found to be linear in the range of 1-5 μg/ml for AC and 5-80 μg/ml for FB. Linearity of AC and FB was plotted by a graph of response factor versus concentration. The correlation coefficient ‘r’ values (n=6) for both AC and FB were ≥ 0.999. Accuracy of the method was calculated by recovery studies (n=3) at three levels. Standard drug solutions containing drugs in the range 1-5 μg/ml for AC and 16-80 μg/ml for FB of concentration was added to previous analyzed test solution. Amount of drug recovered at each level (n=3) was determined. Percent recovery at each level was calculated. System suitability parameters of AC and FB are given in the Table 1. Table 2 shows data from the recovery study for AC and FB were 100.76 and 100.06 respectively. The sample recovery in the formulation was in good agreement with the label claim. High percentage recovery showed that the method was free from interference of excipients used in formulations. The method was simple and had short runtime of 10 min, which make the method rapid. The results of the study indicate that the proposed HPLC method was simple, precise, highly accurate, specific and less time consuming.

**TABLE 1:** SYSTEM SUITABILITY PARAMETERS

Parameter	Atorvastatin calcium	Fenofibrate
Tailing factor*	1.23	1.08
No. of theoretical plate*	3161	7772.83
Capacity factor*	0.67	4.07
Resolution factor*	6.60	14.54
Calibration range	1-5 μg/ml	16-80 μg/ml

* Each value is the Mean of 6 determinations

**TABLE 2:** RECOVERY STUDIES DATA SHOWING AMOUNT OF DRUG RECOVERED FROM SAMPLE SOLUTION AND AVERAGE RECOVERY

Drug	Tablet Amount (μg/ml)	Amount Added (μg/ml)	Amount Recovered (μg/ml)*	Recovery (%)	Coefficient of Variation
Atorvastatin calcium	1	1	1.04	103.8	
	1	2	2.01	100	
	1	3	3.1	102.3	0.017
	1	4	4.1	101.5	
	1	5	5.1	101.2	
Fenofibrate	16	16	16.05	100.25	
	16	32	32.3	100.65	
	16	48	47.96	99.9	0.0038
	16	64	63.82	99.7	
	16	80	79.90	99.8	

* Each value is the mean of 3 determinations
